# Measuring serum melatonin in postmenopausal women: Implications for epidemiologic studies and breast cancer studies

**DOI:** 10.1371/journal.pone.0195666

**Published:** 2018-04-11

**Authors:** Lisa W. Chu, Esther M. John, Baiyu Yang, Allison W. Kurian, Yasaman Zia, Kai Yu, Sue A. Ingles, Frank Z. Stanczyk, Ann W. Hsing

**Affiliations:** 1 Cancer Prevention Institute of California, Fremont, California, United States of America; 2 Stanford Cancer Institute, Stanford University School of Medicine, Stanford, California, United States of America; 3 Department of Health Research and Policy (Epidemiology), Stanford University School of Medicine, Stanford, California, United States of America; 4 Department of Medicine, Stanford University School of Medicine, Stanford, California, United States of America; 5 Division of Cancer Epidemiology and Genetics, National Cancer Institute, National Institutes of Health, Bethesda, Maryland, United States of America; 6 Department of Preventive Medicine, Keck School of Medicine, University of Southern California, Los Angeles, California, United States of America; 7 Department of Obstetrics and Gynecology, Keck School of Medicine, University of Southern California, Los Angeles, California, United States of America; 8 Stanford Prevention Research Center, Stanford University School of Medicine, Stanford, California, United States of America; University of South Alabama Mitchell Cancer Institute, UNITED STATES

## Abstract

**Background:**

Circulating melatonin is a good candidate biomarker for studies of circadian rhythms and circadian disruption. However, epidemiologic studies on circulating melatonin are limited because melatonin is secreted at night, yet most epidemiologic studies collect blood during the day when melatonin levels are very low, and assays are lacking that are ultrasensitive to detect low levels of melatonin reliably.

**Objective:**

To assess the performance of a refined radioimmunoassay in measuring morning melatonin among women.

**Methods:**

We used morning serum samples from 47 postmenopausal women ages 48–80 years without a history of breast cancer who participated in the San Francisco Bay Area Breast Cancer Study, including 19 women who had duplicate measurements. The coefficient of variation (CV) and intraclass coefficient (ICC) were estimated using the random effect model.

**Results:**

Reproducibility for the assay was satisfactory, with a CV of 11.2% and an ICC of 98.9%; correlation between the replicate samples was also high (*R* = 0.96). In the 47 women, serum melatonin levels ranged from 0.6 to 62.6 pg/ml, with a median of 7.0 pg/ml.

**Conclusion:**

Our results suggest that it is possible to reliably measure melatonin in postmenopausal women in morning serum samples in large epidemiologic studies to evaluate the role of melatonin in cancer etiology or prognosis.

## Introduction

In 2007, the International Agency for Research on Cancer classified shift work involving circadian disruption as a probable carcinogenic agent to humans (group 2A) [1], based primarily on experimental and observational studies of breast cancer [2]. It is well known that one adverse effect of shift work is the disruption of the worker’s sleep-wake cycle [3]. In humans, the sleep-wake cycle is regulated mainly by melatonin, a hormone produced by the pineal gland in response to low light conditions [4]. Circulating melatonin exhibits a circadian rhythm with highest levels at night, moderate levels in the morning, and lowest levels in the afternoon. Thus, melatonin in the blood is a good biomarker of the circadian clock.

Traditionally, in clinical studies of sleep, melatonin is measured in serum from night time blood draws or in first-void morning urine samples as melatonin’s major metabolite 6-sulfatoxymelatonin in urine is a good indicator of nighttime circulating melatonin levels. However, in most large-scale epidemiologic studies, and particularly in prospective studies, blood samples are usually drawn during the day, and few studies collected urine samples. Due to the much lower concentration of melatonin in serum collected during the day compared with serum collected at night, serum melatonin assays need to have a higher sensitivity to detect the much lower levels in blood. Therefore, to help expand serum-based epidemiologic studies on melatonin, we previously refined a melatonin radioimmunoassay (RIA) for serum-based studies and showed that the assay is reliable in assessing morning melatonin levels in men [5]. In a subsequent study, we showed that there was a high 5-year correlation of melatonin levels, which implies that a single measurement is sufficient to examine associations between melatonin and risk of cancer in epidemiologic studies [6]. However, since there is an association between circulating levels of sex hormones and melatonin in women, it is difficult to extrapolate the findings from men to women, especially for determining the possible range for morning levels of serum melatonin in women [7–9]. In this methodologic study, we extended our investigation to postmenopausal women and assessed the utility of the refined melatonin RIA in measuring circulating levels of morning melatonin.

## Materials and methods

### Study samples

Postmenopausal women included in this study were participants in the San Francisco Bay Area Breast Cancer Study, a population-based case-control study of breast cancer; the parent study recruited cases aged 35–79 from the San Francisco Bay Area diagnosed with a first primary histologically confirmed invasive breast cancer between 1995 and 2002, and controls identified by random-digit dialing [10, 11]. The biospecimen component of the parent study was initiated in 1999 [12]. All participants of the parent study provided signed informed written consent to be included in both components of the study. For the current study, we included non-Hispanic white postmenopausal women without a history of breast cancer (controls) who had provided a blood sample. Because serum levels of melatonin decline during the day, we limited subjects to those with morning blood sample. Fasting blood was collected before 10AM for most study participants. We randomly selected 10 women from each of five age groups (<60, 60–64, 65–69, 70–74, and 75–80 years). To assess reproducibility, we selected 20 of the women from whom we obtained duplicate samples from the same blood draw that were aliquotted at the same time. This study was approved by the Institutional Review Board from the Cancer Prevention Institute of California.

### Serum melatonin assay

Details of the refined melatonin RIA have been described previously [5]. Briefly, the Buhlmann Melatonin RIA kit (ALPCO, Salem, NH) was used with a preceding extraction step for measuring serum melatonin. For each assay, we used 0.5 ml of serum. Each serum sample was first purified using reversed-phase extraction columns, dried, and reconstituted in assay buffer. Melatonin standards for the standard curve, ranging from 0.5 to 50 pg/ml, were also reconstituted in the assay buffer. The samples and standards were then incubated with a highly specific anti-melatonin antibody and iodinated melatonin for 20 h at 4°C, precipitated for the antibody-bound fraction using a solid-phase second antibody for 15 min at 4°C, and centrifuged. The unbound fraction was aspirated and the antibody-bound fraction of iodinated melatonin was counted using a gamma counter. The analytical sensitivity of the assay is 0.5 pg/ml, and the interassay coefficient is 13.0% and 8.3% at melatonin concentrations of 2.4 and 22.2 pg/ml, respectively.

### Assessment of assay reproducibility

Measurements from 47 women, including duplicate measures from 19 women, were used to assess assay reproducibility; three women were excluded from analysis due to reasons stated below (see Results). We used two measures of reproducibility to determine the precision of the melatonin assays: the coefficient of variation (CV) and the intraclass correlation coefficient (ICC). The CV, expressed as a percent, is defined as 100 times the ratio of the SD to the mean: CV = 100 x σ/μ. Small CVs mean less dispersion from the mean and thus better reproducibility. The ICC, expressed as a percent, is calculated as 100 times the ratio of the variance associated with subjects (σ_α_^2^) to the sum of all variances [associated with subjects and other factors such as measurement error (σ_α_^2^+ σ_ε_^2^): ICC = 100 x σ_α_^2^/(σ_α_^2^+ σ_ε_^2^)]. We performed the natural logarithm transformation of the melatonin measures, and used the random effect model to estimate σ_α_^2^ and σ_ε_^2^ (in log scale), with the adjustment of age. Following the method by Gail *et al* [13], the CV (in its original scale) was estimated by σ_ε._ Assays with CVs <20% and ICCs >80% are usually considered reasonable. Pearson’s correlation coefficient (R) was used to measure the correlation between the duplicate serum samples from the same subject.

### Associations between melatonin level and selected subject characteristics

We used data from the study to identify possible correlations between morning serum melatonin levels and time of blood draw, age at blood draw, reason for menopause (natural or surgical), hormone therapy (HT) use at the time of interview, duration of HT use at the time of interview, and body mass index (BMI; weight divided by height squared expressed in kg/m^2^, measured at blood draw). Range, medians, as well as geometric means and 95% confidence intervals (CIs) of morning serum melatonin levels were calculated for each subgroup of women. For subjects with duplicate measurements, the mean of the replicate measurements was used in the analyses. We performed the analysis of covariance to calculate the p value for the association between each variable and melatonin level, with mutual adjustment of each other. P values < 0.05 were considered statistically significant.

## Results

Of the 50 women selected for the study, two were excluded from analysis because of undetectable melatonin levels and one was excluded because it was later determined that the blood draw occurred after 12:00 pm. Thus, data from 47 women were used for analysis.

As shown in **Table 1**, we found that the assay was reliable in detecting melatonin in morning serum, with a CV of 11.2%. The correlation between duplicate samples was high (R = 0.96). The ICC was 98.9% after adjusting for age. Further adjustment of other covariates including BMI, time of blood draw, menopausal type and hormone therapy use did not materially change the results (data not shown).

**Table 1:** Range of morning melatonin levels and reproducibility of serum melatonin assays in postmenopausal non-Hispanic white women.

**Serum Melatonin Levels in Postmenopausal Women**	
N	47
Age Range (years)	
Range	48–80
Median	67
Mean	67.2
Time of Blood Collection	
Range	6:50 AM-10:38 AM
Median	8:40 AM
BMI, kg/m^2^	
<25	20 (54%)
25–29.9	15 (32%)
≥30	12 (26%)
Reason for Menopause	
Natural	39 (83%)
Surgical	8 (17%)
Hormone Therapy Use	
Never	4 (9%)
Past	32 (68%)
Current	11 (23%)
Duration of Hormone Therapy Use (years)	
0	4 (9%)
<10	17 (36%)
10–19.9	15 (32%)
≥20	11 (23%)
Melatonin (pg/ml)	
Range	0.6–62.6
Median	7.0
Interquartile Range	3.1–13.0
Geometric Mean (95% CI)	6.7 (4.9–9.1)
**Reproducibility of the Melatonin Assay**	
N	19
Replicates per sample	2
CV (%) *	11.2
ICC (%)*	98.9
Correlation (*R*)	0.96

* Adjusted for Age

Abbreviations: BMI, body mass index; CI, confidence interval; CV, coefficient of variation; ICC, intraclass correlation coefficient

**Fig 1:**
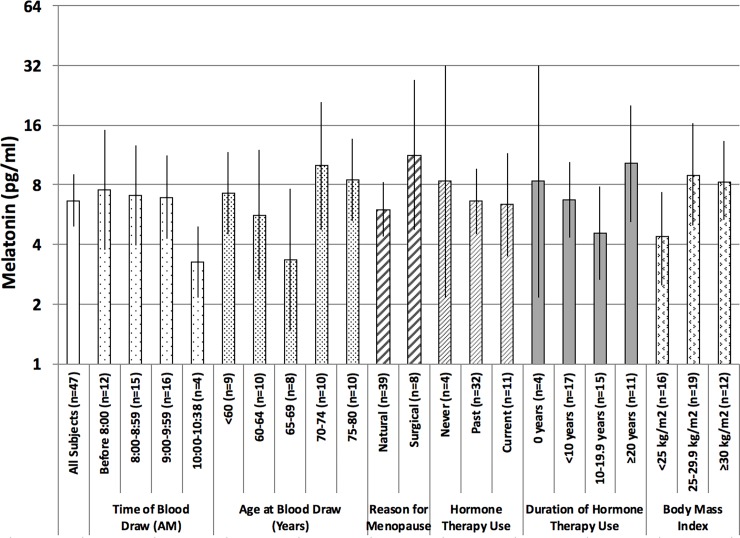
Morning melatonin levels in non-Hispanic white postmenopausal women, stratified by time of blood draw, age group at blood draw, reason for menopause, hormone therapy use, duration of hormone therapy use, and body mass index.

Among the 47 postmenopausal women, morning serum melatonin levels ranged from 0.6 to 62.6 pg/ml (median of 7.0 pg/ml and geometric mean of 6.7 pg/ml; **Table 1**). As expected, serum melatonin levels decreased as the morning progressed, with higher levels found in the earlier part of the morning (before 10:00 AM; **Fig 1**). Although the mean age and time at blood draw were similar among the groups of women defined by type of menopause (data not shown), there was suggestive evidence for differences in melatonin levels between women with a natural menopause (n = 39; geometric mean of 6.0 pg/ml) versus those with surgical menopause (n = 8; geometric mean of 11.3 pg/ml). Higher melatonin levels were seen in women who were overweight (BMI 25–29.9 kg/m^2^; geometric mean of 8.4 pg/ml) or obese (BMI ≥30 kg/m^2^); geometric mean of 7.3 pg/ml) compared to women who had a BMI in the normal range (BMI <25 kg/m^2^; geometric mean of 5.3 pg/ml). There were no clear trends in morning serum melatonin levels by age, HT use or duration of HT use. None of the associations assessed above were statistically significant (p ≥ 0.05).

## Discussion

Similar to our previous study in men, we confirmed that using the refined melatonin assay, serum melatonin can be reliably measured in postmenopausal women in blood samples collected in the morning [5, 6]. To our knowledge, this is the first study to examine the validity of morning melatonin measurements in postmenopausal women. Our results suggest that it is possible to use morning serum for studies that investigate the role of circulating melatonin in cancer etiology in postmenopausal women. Further refinement of the assay to use smaller serum volumes would be desirable.

The range of morning serum melatonin in postmenopausal women is wider than that reported in our previous study in men (35 Caucasian American men, 50–81 years of age, morning melatonin levels between 1.4 and 34.3 pg/ml) [5]. Possible reasons for the observed differences in circulating melatonin levels between men and women include: 1) differences in populations between the two studies; 2) small sample sizes; and 3) gender differences in circulating levels of sex hormones, which have been shown to influence nocturnal melatonin levels [14]. Larger studies are needed to confirm whether there are true gender differences in circulating morning melatonin levels. Future studies should also include women from racial/ethnic groups other than non-Hispanic white.

We did not see clear associations between menopausal type or duration of HT use and morning melatonin levels. This is consistent with a recent clinical trial, which showed that although HT use delayed the peak time of nocturnal melatonin secretion similar to the pattern seen with exposure to light at night, HT had no effect on serum melatonin levels [15]. In our study, women with surgical menopausal had higher melatonin levels than women with a natural menopause, despite being of similar age and having blood drawn at similar times. Larger studies are needed to confirm this finding. Future studies should also evaluate whether time since onset of menopause is related to morning serum melatonin levels. In addition, since previous studies have shown that postmenopausal women have lower nighttime serum melatonin levels than perimenopausal women [14], future studies should include peri- and pre-menopausal women to determine whether our findings can be extended to women of all ages.

Our study showed suggestive positive associations between BMI and morning melatonin levels. Our result is inconsistent with previous studies in women measuring 6-sulfatoxymelatonin, the primary metabolite of melatonin [16–20]. However, previous studies either included both pre- and postmenopausal women [16–18] or women who were younger than those in the current study [19, 20], which renders direct comparisons to the current study difficult. In studies of men, BMI correlated positively with melatonin levels [6, 21]. Additional larger studies are needed to clarify the association between BMI, menopausal status, and morning serum melatonin levels.

Given the evidence suggesting that circadian rhythm disruptions are involved in the etiology of several cancers, including breast cancer [22–24], epidemiologic studies should investigate whether melatonin, as a key intermediary of chronodisruption [25], influences cancer risk. Future epidemiologic studies of serum melatonin and cancer should take into consideration or adjust for possible determinants of circulating melatonin. Our study provides some insights into factors associated with morning melatonin in postmenopausal women. However, due to our relatively small sample size, larger studies are needed to confirm or refute some of our findings. Other factors should also be evaluated. For instance, exposure to light at night has been shown to delay the peak time of nocturnal melatonin secretion, which results in a shift in the overall profile of circulating melatonin, including levels in the morning hours [26–30]. Whether any of these determinants of circulating melatonin influence the association between melatonin and cancer warrants further investigation.

## Supporting information

S1 Dataset MelatoninData_PlosOne upload new. (XLS)

## Abbreviations

BMI: body mass index
CV: coefficient of variation
HT: hormone therapy
ICC: intraclass coefficient
R: correlation coefficient
RIA: radioimmunoassay
SE: standard error
